# Around the Hospitals

**Published:** 1892-10-15

**Authors:** 


### Around the Hospitals.

The **Richmond Hospital** is a modest little institution. A children's ward and more accommodation for the nursing staff have been necessary for some time. The public up to March last had not been appealed to, owing to a fear that very pressing local needs made the authorities fearful lest the appeal would meet with but poor response. We hope soon to learn that they have taken courage, and confident of success are reaping the harvest the merits of the case deserve. The last improvement in the arrangements of the hospital has been the reconstruction of the ventilation system in the wards, which has proved of great benefit. The institution is under distinguished patronage, nor are the names on the list of patrons merely ornamental, very real interest being taken in the hospital. A balance to the good is apparent in the accounts proves this fact, and should encourage the management to proceed towards all improvements necessary in the best interests of the institution. Subscribers may be equally satisfied that the money entrusted to the authorities is used with care, and this has never been more apparent than during the past year, a glance at the accounts showing that the expenditure has been less than in the previous year, whilst the number of patients have been larger, and in no year has the cost per bed been so small as during 1891. One excellent rule of the institution is that referring to domestic servants. It provides that servants shall be paid for at the rate of 10s. 6d. a week when sent in by subscribers, and 12s. 6d. a week when this is not the case. Frequently contributors of perhaps but £2 2s. per annum will expect their servants to be received for a considerable period without further payment on their part. The Richmond Hospital also provides against the retention of lengthy cases under one letter of admission by rendering such available for six weeks residence only.

The **Stockton Hospital** has made good use of its increased accommodation, and during the present year 147 in-patients above the number received the previous year have been admitted. The work of the hospital has further been rendered more complete by the establishment in Stockton of a branch of the London Surgical Aid Society, through which patients of the hospital will be especially benefited. The plan of one penny per week subscriptions from the poorer classes has answered admirably with regard to the feeling of this section of the community towards the institution, as thereby a feeling of proprietorship and participation in the good work is engendered. The Stockton Hospital is one of the few fortunate institutions which hold a substantial balance in hand.

The **Great Ormond Street Hospital for Children,** always a popular institution with the public, is now on its way to extend its work. The buildings in hand during the past year are not yet completed, as to furniture and fittings, and the whole of the building fund has been exhausted. Large sums are still required to complete all the arrangements. The ordinary expenses have to a great extent been covered, and the festival dinner in March, over which the Duke of Fife presided, greatly assisted towards this end. The institution has been fortunate in legacies during the past year, and has also experienced an increase in donations and subscriptions. The latter, it is hoped, will continue to multiply, as the additions to the hospital will of course increase the expenditure. During 1891 two new cots were founded, one the "Grassendale Cot," and the other the "Harry Howard." During the year, 1,248 in-patients were treated, and 248 children enjoyed the benefit of the Convalescent Home at Highgate.

